# Food Choice Motives When Purchasing in Organic and Conventional Consumer Clusters: Focus on Sustainable Concerns (The NutriNet-Santé Cohort Study)

**DOI:** 10.3390/nu9020088

**Published:** 2017-01-24

**Authors:** Julia Baudry, Sandrine Péneau, Benjamin Allès, Mathilde Touvier, Serge Hercberg, Pilar Galan, Marie-Josèphe Amiot, Denis Lairon, Caroline Méjean, Emmanuelle Kesse-Guyot

**Affiliations:** 1 Sorbonne Paris Cité Epidemiology and Statistics Research Center (CRESS), Inserm U1153, Inra U1125, Cnam, Paris 13 University, Nutritional Epidemiology Research Team (EREN), F-93017 Bobigny, France; s.peneau@eren.smbh.univ-paris13.fr (S.P.); b.alles@eren.smbh.univ-paris13.fr (B.A.); m.touvier@eren.smbh.univ-paris13.fr (M.T.); s.hercberg@eren.smbh.univ-paris13.fr (S.H.); p.galan@eren.smbh.univ-paris13.fr (P.G.); c.mejean@eren.smbh.univ-paris13.fr (C.M.); e.kesse@eren.smbh.univ-paris13.fr (E.K.-G.); 2 Department of Public Health, Hôpital Avicenne, F-93300 Bobigny, France; 3 Aix Marseille Université, Nutrition Obésité et Risque Thrombotique (NORT), INRA 1260, INSERM, UMR S 1062, 13005 Marseille, France; marie-josephe.amiot-carlin@univ-amu.fr (M.-J.A.); denis.lairon@orange.fr (D.L.)

**Keywords:** food choice motives, organic food consumption, sustainability, clusters of consumers

## Abstract

The purpose of this study was to examine food choice motives associated with various organic and conventional dietary patterns among 22,366 participants of the NutriNet-Santé study. Dietary intakes were estimated using a food frequency questionnaire. Food choice motives were assessed using a validated 63-item-questionnaire gathered into nine food choice motive dimension scores: “absence of contaminants”, “avoidance for environmental reasons”, “ethics and environment”, “taste”, “innovation”, “local and traditional production”, “price”, “health” and “convenience”. Five consumers’ clusters were identified: “standard conventional food small eaters”, “unhealthy conventional food big eaters”, “standard organic food small eaters”, “green organic food eaters” and “hedonist moderate organic food eaters”. Relationships between food choice motive dimension scores and consumers’ clusters were assessed using analysis of covariance (ANCOVA) models adjusted for sociodemographic factors. “Green organic food eaters” had the highest mean score for the “health” dimension, while “unhealthy conventional food big eaters” obtained the lowest mean score for the “absence of contaminants” dimension. “Standard organic food small eaters”, “green organic food eaters” and “hedonist moderate organic food eaters” had comparable scores for the “taste” dimension. “Unhealthy conventional food big eaters” had the highest mean score for the “price” dimension while “green organic food eaters” had the lowest mean scores for the “innovation” and “convenience” dimensions. These results provide new insights into the food choice motives of diverse consumers’ profiles including “green” and “hedonist” eaters.

## 1. Introduction

Although sustainability is not only a matter of consumer choice, consumers have a key role to play in the major challenge of achieving sustainable food system and healthy diets [1,2]. By shifting their eating practices towards more environmentally friendly and healthier habits, consumers can contribute to change the demand [2,3,4,5,6,7] and encourage the development of policy interventions aiming at improving the supply [8]. Their food choices are therefore crucial in the transformation towards sustainable diets as defined by the Food and Agriculture Organization of the United Nations (FAO) [1].

Numerous studies on food choice motives have been carried out. To comprehensively address the broad diversity of determinants of food choice motives, the “Food Choice Questionnaire” developed by Steptoe et al. [1] in a demographically heterogeneous UK sample has been widely used and adapted thereafter [2]. Nine dimensions underlying food choices were identified: “convenience”, “price”, “health”, “sensory appeal”, “weight control”, “natural content”, “mood”, and “familiarity” and “ethical concerns”. Food supply and related-information have evolved very rapidly and, over the last few years, higher concerns for sustainability in food choices have been observed, especially among certain consumer subgroups [3]. Even though taste, healthiness and price [1,2,4,5] remain the strongest drivers of food choice, environmental considerations are now important determinants of food choices too, in particular for certain segments of consumers [3]. A research, based on a specifically developed tool emphasizing on sustainable concerns (including local and traditional production aspects), was therefore tested on a sample of the NutriNet-Santé from the French population (*n* = 1000) and allowed to identify new dimensions associated with consumers’ food-buying motives [6]. This questionnaire was validated and enabled to assess dimensions related to food choice motives with a particular focus on dimensions of sustainability.

In this context of emergence of new concerns about sustainability and health issues, the organic food market represents a growing and dynamic sustainable market. Thus, in France in 2014, sales of organic products have totaled 5.5 billion euros revealing a 10 percent increase from the previous year [9]. Studies carried out in the French cohort NutriNet-Santé about organic food consumers underlined specific eating habits among high organic food consumers including vegetal-based dietary patterns [10] although different types of eater profiles emerged [11].

Research on motivations of organic food consumers are plentiful [12,13,14,15,16,17,18,19,20,21]. The range of motivations covers both ethical or environmental aspects [15,16,17,18,21] as well as more self-centered considerations such as health or sensory aspects [15,17,18,19,20]. In France, taste and traditional characteristics are also important factors [22] as well as freshness or naturalness [23].

Furthermore, it seems that purchase motivations depend on the degree of commitment of the individual in the organic dynamics [12,23,24]. According to some studies, regular consumers are mostly motivated by ethical reasons, whereas for occasional buyers health considerations remain the main driving factors [18,25]. A survey reported in a Swedish report indicated that environmental motives were particularly important among young consumers [26]. Another study [27] revealed that regular and occasional consumers had both high concerns for environment.

Moreover, it has also been advanced that food choice motives may vary across food categories. The reasons frequently cited by consumers for consuming fruit and vegetables are health, taste or provenance while, for instance, concerning pork meat, origin as well as prices and sale promotions are also important factors when purchasing [28]. Similarly, some categories of organic products are consumed for health reasons. For instance, chemicals in foods are a major concern for organic fruit and vegetables while this is less the case for organic dairy products [24].

Research has mainly focused on motivations for choosing organic food among frequent buyers vs. non-buyers while it is crucial to address the level of importance of various food choice motives across different types of consumers with various behavior patterns as regards organic food to highlight consumers’ trade-offs when purchasing.

The purpose of this study was thus to examine, on a large scale, using an epidemiological approach, the food choice motives of consumers’ clusters characterized by different organic and conventional food patterns based on detailed and accurate data on organic food consumption and food choice motives. We also paid particular attention to the relative importance of food choice motives according to food categories.

## 2. Materials and Methods

### 2.1. Data Collection

#### 2.1.1. Population

Data were collected by web-based questionnaires from the NutriNet-Santé cohort study. The NutriNet-Santé study is an observational prospective open cohort study, launched in 2009 in France which aims at investigating the relationships between nutrition and health as well as the determinants of eating behaviors and nutritional status [29]. Participants complete a set of self-administered web-based questionnaires assessing sociodemographic, lifestyle, dietary characteristics and health and anthropometric status. All baseline questionnaires were first pilot-tested and compared with traditional administration methods [30,31,32,33]. As part of their follow-up, participants are also regularly invited to fill in optional questionnaires pertaining to determinants of dietary behaviors, diet- and health-related factors.

#### 2.1.2. Ethics Approval and Consent to Participate

This study was conducted according to guidelines laid down in the Declaration of Helsinki and was approved by the International Research Board of the French Institute for Health and Medical Research (IRB Inserm No. 0000388FWA00005831) and the “Commission Nationale Informatique et Libertés” (CNIL No. 908450 and No. 909216). Electronic informed consent was obtained from all participants. This study is registered in EudraCT (No. 2013-000929-31).

#### 2.1.3. Dietary Data: Assessment of Organic and Conventional Intakes

To estimate organic and conventional food consumption, a self-administered web based semi-quantitative Organic-Food Frequency Questionnaire (Org-FFQ) was used. The Org-FFQ has been extensively described elsewhere [34]. Briefly, the Org-FFQ was based on a validated semi-quantitative FFQ [35] to which has been added a 5-point type Likert frequency scale to estimate the frequency of organic food consumption. It consisted of 264 beverage and food items with standard serving sizes and a drop-down list of 4 frequency categories (per day, per week, per month and per year). Participants were asked to report their frequency consumption and the amount of foods consumed over the previous year. For each food item, participants were also asked the following question: “how often was the product from organic origin?”. They had to choose one of the five following modalities: never, sometimes, half of the time, often and always.

#### 2.1.4. Assessment of Food Choice Motives

Data regarding food choice motives were collected using a validated questionnaire specifically developed in the NutriNet-Santé study to measure food choice motives including sustainable concerns during purchasing [36].

The questionnaire consisted of 63 items divided into two main sections: one focused on general aspects of food purchasing and the second focused on purchase of specific food groups (meat/fish/fruits and vegetables/dairy products). All questions in the first section were formulated as follows: “When I purchase food, I take into account...”. For each food group of the second section, participants were asked whether they bought this food group. If so, they answered all questions concerning that food group. If not, they answered specific questions on reasons for not buying this food group. Questions were worded as follows: (i) “When I purchase (meat/fish/fruits and vegetables/dairy products), I take into account...” for all participants; (ii) “I purchase (meat/fish/fruits and vegetables/dairy products) for health/taste/etc. issues” only for participants who reported purchasing that food group; and (iii) “I avoid purchasing (meat/fish/fruits and vegetables/dairy products) for environmental/price/etc. issues” for participants who did or did not purchase that food group. For each statement, participants were asked to rate their level of agreement on a 5-point Likert scale from “I strongly disagree” to “I strongly agree”. Respondents could indicate that they did not know.

The questionnaire was internally validated using factor analysis. The first-order analysis enabled to identify the nine following food choice dimensions: (1) absence of contaminants; (2) environmental limitations or avoidance for environmental reasons; (3) ethics and environment; (4) taste; (5) innovation; (6) local and traditional production; (7) price; (8) health; and (9) convenience. The “avoidance for environmental reasons” dimension can be considered as stronger than the motivation “ethics and environment” as it implies a radical commitment for conserving the environment. A second-order factor analysis was conducted to determine whether factors with high inter-correlations in the first-order factor confirmatory factor analysis were influenced by a broader dimension. A second-order factor was therefore highlighted “healthy and environmentally friendly consumption” which included four first-order sub-dimensions (“ethics and environment”, “local and traditional production”, “health” and “absence of contaminants”). The underlying motives identified overlapped on many aspects with the previous validated Food Choice Questionnaire [37] (including the identification of motives pertaining to taste, price, ethical concerns or convenience) but also permitted to highlight new dimensions including “avoidance for environmental reasons”, “local and traditional production” and “innovation” [37]. Given each factor consisted of different number of items (from 4 to 18 items), for comparative purposes, we used weightings, in order to standardize ratings to obtain score values ranging from 0 (no concern) to 10 (strong concern). Table 1 describes the questionnaire items included in each dimension.

In addition, we also provided radar diagrams presenting the scores for five food choice motives (ranging from 0 to 10) for fruit and vegetables, dairy products and meat (derived from the set of questions pertaining to health, taste, environment, price and avoidance for each food group) across the five clusters.

### 2.2. Statistical Analysis

#### 2.2.1. Selection of the Study Sample

The Org-FFQ, an optional questionnaire, was administered to 104,080 participants of the NutriNet-Santé cohort in June 2014 over a period of 5 months. A total of 33,384 participants completed the questionnaire (i.e., 32% of the invited cohort participants). Within this sample, we included individuals who were not energy under- or over-reporters (*n* = 31,287), with no missing covariates (*n* = 28,967) and not living overseas (*n* = 28,245). Among them, we selected those who had completed the questionnaire of food choice motives, leaving 22,366 individuals for the present study. To identify under- and over-reporting participants, we compared individual energy requirement using basal metabolic rate by Schofield’s equations [38] with energy intake and excluded individuals with ratios below or above cut-offs (0.35 and 1.93) previously determined [34]. We also compared non-respondents to the Org-FFQ respondents using chi-square test and Student *t*-test.

#### 2.2.2. Characteristics of the Participants

Compared to non-respondents, respondents to the Org-FFQ were less often women (79% vs. 74%), more likely to be retired (14% vs. 36%) and older (44.62 ± 14.20 years vs. 53.20 ± 14.07 years). Characteristics of the participants are presented in Table 2. More than ¾ of the participants were female. The mean age was 53.7 ± 13.6 years old. The participants reported on average a proportion of 0.28 ± 0.26 of organic food in the diet and a total energy intake of 1989 ± 627 kcal/day. More than half of the sample held a high school diploma. Almost 30% of the participants reported a monthly income per household unit higher than 2700 euros. Regarding place of residence, 22% of the participants resided in a rural community and 44% in an urban unit with a population higher than 200,000 inhabitants.

#### 2.2.3. Estimation of Dietary Intakes

Daily food intakes in grams were calculated by multiplying the consumption frequency of each food by the quantities consumed. The procedure aiming at estimating organic food intake has been previously described [34]. Briefly, for each item, organic consumption was estimated by multiplying the consumption by the following weightings 0, 0.25, 0.50, 0.75 and 1, allocated to the respective frequencies “never”, “rarely”, “half of the time”, “often” and “always” [34].

The proportion of organic food in the diet was computed by dividing the total organic food intake (g/day) out of the total food intake (g/day) excluding water. To assess daily energy intake, the original NutriNet santé food composition table [39] was used.

#### 2.2.4. Cluster Analysis

In order to identify subgroups of consumers, we also performed a principal component analysis (PCA). The PCA was applied to 16 organic food categories (g/day) and 16 corresponding conventional food categories (g/day) and was followed by a two-step cluster analysis (a Ward’s hierarchical ascendant classification followed by a k-means clustering for stabilization purpose) based on the first six dimensions retained in the PCA using as criteria: eigenvalues >1, scree-test and interpretability of factors. It allowed identifying 5 clusters of consumers with similar dietary profiles. Clusters were labeled according to their major dietary traits (Table 3). Five clusters were identified and labeled as follows: “standard conventional food small eaters” (characterized by low intake and low consumption of organic food products), “unhealthy conventional food big eaters” (characterized by high intake and a very low consumption of organic food products), “standard organic food small eaters” (characterized by low intake and high consumption of organic food products), “green organic food eaters” (characterized by a very high consumption of organic food products and high intake of plant-based foods), and “hedonist moderate organic food eaters” (characterized by high intake of alcohol and a moderate consumption of organic food products).

Regarding sociodemographic characteristics (data not tabulated), “unhealthy conventional food big eaters” and “hedonist moderate organic food eaters” comprised the highest percentages of men while “green organic food eaters” included the highest percentage of post-secondary graduate individuals.

#### 2.2.5. Statistical Modeling

Mean food choice dimension scores and 95% Confidence Intervals (95% Confidence Intervals (CI)) across clusters adjusted for sex, age, educational level and household income were assessed using analysis of covariance (ANCOVA). We also provided findings from post-hoc tests for differences in means. We used Tukey’s method to account for multiple comparisons.

All tests were two-sided. All analyses were performed using SAS package version 9.4 (The SAS Institute Inc., Cary, NC, USA).

## 3. Results

### 3.1. Food Choice Motive Dimension Scores

The highest mean food choice motive score was found for the “taste” dimension followed by the “health” and “absence of contaminants” dimensions (Table 4). The lowest mean food choice motive score was found for the “avoidance for environmental reasons” dimension followed by the “innovation” dimension.

### 3.2. Food Choice Motives across Clusters of Consumers

Mean food choice dimension scores across clusters of consumers are presented in Table 5.

“Green organic food eaters” had the highest mean scores for the “absence of contaminants” and “health” dimensions, while “unhealthy conventional food big eaters” obtained the lowest mean score for the “absence of contaminants” dimension. “Hedonist moderate organic food eaters” and “unhealthy conventional food big eaters” obtained the lowest mean scores for the “health” dimension. Regarding the “avoidance for environmental reasons” dimension, the lowest mean score was observed among “unhealthy conventional food big eaters”. “Green organic food eaters” were the most concerned by the “ethics and environment” and “local and traditional production” dimensions when choosing foods while “unhealthy conventional food big eaters” were the least concerned by these aspects. In particular, “green organic food eaters” had a prominent score for the overall dimension “healthy and environmentally friendly consumption”.

“Standard organic food small eaters”, “green organic food eaters” and “hedonist moderate organic food eaters” had comparable scores for the “taste” dimension. “Unhealthy conventional food big eaters” had the highest mean score for the “price” dimension and “green organic food eaters” had the lowest. “Green organic food eaters” had the lowest mean scores for the “innovation” and “convenience” dimensions.

### 3.3. Food Choice Motives According to Food Categories across Clusters

Overall, whatever the food category (Supplementary Materials Figure S1), similar mean scores for “taste” were observed across clusters. Regarding “environment”, lower means were observed for dairy products, while mean scores for “price” were slightly higher for meat. Higher scores for “avoidance for environmental reasons” were allocated to meat compared to other food groups, especially among “green organic food eaters”. As regards “health”, the highest score was observed for fruit and vegetables and the lowest for dairy products across all clusters. Globally, for a specific food group, the different clusters prioritized the same motives (same order) but the level of importance varied across clusters (for instance, overall, higher scores were found among “green organic food consumers” than for other clusters).

## 4. Discussion

Using detailed information on organic and conventional food intakes and motives for choosing foods in a large population of French adults, we showed that the importance of food choice motives varied across clusters of organic and conventional consumers. Overall, taste was a strong motivator for food choice whatever the type of consumers. Health and ethics/environmental concerns were more important drivers of food choice motives in all profiles of organic food consumers with respect to their conventional counterparts. This was the opposite for price, innovation and convenience.

Consistent with previous studies documenting the importance of healthiness and food safety for consumers of organic food [12,40,41,42,43,44], “green organic food consumers” and “standard organic food small eaters” highly valued the absence of contaminants in their food choice motives. The same was true, but to a lesser extent, regarding the “health” dimension that included health impact and nutritional values of food. These findings are in line with the idea that, for organic food consumers, the health facets are essential in their food choices [12,40,41,42,43,44]. “Unhealthy conventional food big eaters” and “hedonist moderate organic food eaters” had the lowest ratings for the “health” dimension. This was in accordance with their unhealthier dietary patterns that included high consumption of sweet products for the first and high consumption of meat and alcohol for the second. These two clusters of consumers, who exhibited specific sociodemographic profiles, may be less interested in long-term effect of food consumption on health/diseases, and thus do not focus on this aspect when purchasing foods.

Our study also clearly showed that “green organic food consumers” were more motivated by ethical choices than their conventional counterparts. This is consistent with previous studies reporting that ethical considerations are core values among consumers of organic foods and more generally among sustainability-concerned consumers [12,16,20,40,42,43,45,46,47].

In this study a second order factor was considered, that encompassed two main pillars of diet sustainability as described by FAO (i.e., health and environment) [1]. “Green organic food eaters” obtained the highest score for this high-order dimension. In a recent work, Aschemann-Witzel et al. [7] pointed out that in consumers, health and environment dimensions are not mutually exclusive and are part of the same concern for a healthier and more ecological lifestyle (“healthier body and planet”). Therefore, the altruistic and selfish motivations that drive consumers when choosing foods may be interconnected and should be taken in consideration together when promoting sustainable food consumption.

In our study, we also observed that “green organic food eaters” and “standard organic food small eaters” had the lowest scores regarding “convenience” and “innovation” dimensions. This may be related to the diffidence of organic food consumers towards agro-biotechnology. A study thus showed that consumers who considered food biotechnology as a risk factor were more prone to buy organic products [48]. These findings may also be related to the measure of the convenience in our study, which referred to ease of use (rapid preparation, easy to cook). Cooking convenience might be related to processed food and specific “unnatural” cook practices (such as the use of a microwave or ready-made products) while freshness and naturalness have been shown to be good predictors of organic food consumption [23]. The organic food supply is also particularly important for raw foods such as fruit and vegetables or eggs [49]. Another hypothesis may be that for “green organic food consumers”, the “convenience” aspect is of secondary importance compared to the efforts provided when purchasing food (e.g., getting local supplies from small organic food markets rather than the local supermarket).

Few studies have included the “innovation” dimension in their description of food choice motives in particular in relation to organic food consumption. We showed herein that “green organic food consumers” had the lowest score regarding the innovation aspects when choosing foods, reflecting their lack of interest for this topic. This can also be attributed to concerns of these consumers towards processing or industrialization of food. It can also be hypothesized that the term “innovative character” encompasses different aspects. The responses proposed in the questionnaire may have been perceived as related to processing or packaging features. Organic food consumers may be prone to adopt “new” natural products (e.g., superfoods). Qualitative studies based on open questions may help to better understand how different profiles of consumers interpret the terms “convenience” or “innovation” and what they cover, supplemented by questions pertaining to cooking skills [50].

Price has always been shown as essential in food choice motives [37,51,52,53] and cost the major constraint for buying organic products [12,15,46]. In our study, “green organic food eaters” and “standard organic food small eaters” were less concerned by the price when choosing foods than “conventional food big eaters”. This may be firstly explained by the intrinsic sociodemographic characteristics of organic food consumers (generally more educated and with higher income) [12,54,55,56]. However, these results were observed despite the adjustment for income in addition to sex, age and education.

Another finding of our study was that all groups share the “taste” dimension. This motivation was previously shown to be weakly correlated with other dimensions [36]. The importance of sensory appeal for choosing food whatever the social status has been established in the literature [37,51]. This means that public health policies and initiatives aiming to promote sustainable/organic food consumption should ensure a high sensorial quality of sustainable products.

Our findings about the different categories of products corroborate the importance of taste when choosing foods, since we observed similar scores whatever the type of foods, across all profiles of consumers. Price was considered as slightly more important when purchasing meat than dairy products or fruit and vegetables, in line with a qualitative study conducted on pork meat showing that price and promotions (as well as origin of production) were the main drivers when purchasing this type of meat [28]. In our study, participants paid particular attention to the environment when choosing fruit and vegetables. The lowest scores for the health-related motives were found for dairy products. Participants avoided especially meat for environmental reasons. This particularly held for “green organic food consumers”, for whom the “avoidance for environmental reasons” dimension was comparatively high for meat compared to fruit and vegetables or dairy products. This is consistent with the main reasons of avoidance of meat found in other studies [57,58,59]. Motivations for meat eviction involve both the concern for health and the environment. Our research did not permit to understand the underlying beliefs behind food choice motives for choosing specific foods as the questions regarding these aspects were voluntary uncomplicated. However, complementary studies can be carried out to explore in depth the representations associated with factors such as “environment” or “health” across different food groups.

One of the major strengths of our study lies in its large sample size that enabled us to cover a diversity of eating habits. A novel aspect of this study was also the detailed estimation of the organic food intake [34]. It enabled to identify different types of conventional and organic eaters. Furthermore, the evaluation of food choice motives was based on a validated questionnaire which specifically focused on sustainability [36]. Some motives rarely described in the literature have been included in our study, in particular the following dimensions: “avoidance for environmental reasons”, “local and traditional production” and “innovation”, enhancing the description of motivations.

Caution is however needed when extrapolating our results to the general population. Participants were indeed part of a web-based cohort focusing on nutrition and were therefore particularly interested in nutrition-related issues. In addition, a study comparing sociodemographic characteristics of the NutriNet-Santé participants to national figures showed that the percentages of women and educated individuals were higher in the NutriNet-Santé although the sample exhibited marked geographical and sociodemographic diversity [60]. Another study compared dietary intakes of sample from the NutriNet-Santé to a nationally representative survey and found similar intakes as regards carbohydrates, lipids, protein, and energy but higher intakes of fruit and vegetables and lower intakes of alcohol and nonalcoholic beverages [61]. Furthermore, respondents to the Org-FFQ were older and more often men than non-respondents. Finally, the study sample included a high proportion of women and individuals with higher level of education. These particular profiles have been shown to be key predictors of sustainable consumption [62,63]. This may have led to an over-representation of individuals concerned by sustainability-related issues and in turn to an over-representation of individuals engaged in green consumption. In addition, filling out the questionnaires pertaining to organic food and food choice motives were optional, which may have led to an additional selection bias. Data collection was based on self-administered questionnaires which are prone to measurement errors.

Our findings have underlined that food choice motives differed according to consumers’ profiles. From a public policy perspective, there is a need to develop specific targeted approaches adapted to different profiles. Further research, based on qualitative studies, should address in more detail the coping strategies among the different types of consumers in real conditions as the motives may vary according to the level of commitment of the individuals to the sustainable market [64]. More specifically, we found that certain subgroups seemed already well engaged in sustainable food consumption. Initiatives aiming to foster healthy and sustainable consumption should therefore prioritize the non-engaged consumers, in line with findings from a qualitative study carried out in France and Italy [64]. Furthermore, food choice motives such as “absence of contaminants” or “innovation” were not of equal importance across the different clusters. In this sense, the “self-regulation” theory developed by Higgins [65] which distinguishes self-regulation with a promotion focus (accomplishments and aspirations) from self-regulation with a prevention focus (safety and responsibilities) could be useful to propose public-awareness recommendations consistent with one’s self-regulation. Moreover, to promote sustainable consumption and production, barriers such as price should also be taken into consideration particularly among certain groups of consumers.

As food choice motive may change over time, further research needs to investigate to what extent motivations could affect the healthiness and the sustainability of the diet. What is also noteworthy is, that in our study, organic food consumers paid a lot of attention to the environment, health and safety aspects of foods while the positive impacts of organic food on the environment and health in particular still need to be documented [66,67,68,69,70,71].

## 5. Conclusions

Although not sufficient, sustainable diets are necessary conditions of sustainable food systems [72] and consumers are among the main drivers. As diets beneficial to health may also be beneficial to the planet [2,6,73,74], initiatives that aim at fostering environmentally-friendly and healthy consumption should therefore have a holistic approach by taking into account both sustainability components (i.e., health and environment).

## Figures and Tables

**Table 1:** Overview of the 63 questionnaire items included in the first- and second- order dimensions determined by factor analysis.

Dimension	Questionnaire Item
*First-order dimension*	
Absence of contaminants (5 items)	Exposure to chemicals (F)
	Fishing method (F)
	Additives (D)
	Exposure to chemicals (G)
	Additives (G)
Avoidance for environmental reasons (4 items)	Not buying for environmental reasons (F)
	Not buying for environmental reasons (D)
	Not buying for environmental reasons (M)
	Not buying for environmental reasons (FV)
Ethics and Environment (18 items)	Pollution caused by production (G)
	Respect for human/workers’ rights (G)
	Impact on earth’s resources (G)
	Respect for working conditions (G)
	Production waste (G)
	Pollution caused by transport (G)
	Environmental impact (G)
	Energy expenditure (G)
	Occupational integration (G)
	Fair payment for producers (G)
	Environmental impact (M)
	Environmental impact (FV)
	Amount of packaging (G)
	Fair trade product (G)
	Environmental impact (D)
	Respect of hygiene conditions (G)
	Political values of the country of the food’s origin (G)
	Environmental impact (F)
Taste (4 items)	Taste of food (FV)
	Taste of food (D)
	Taste of food (G)
	Taste of food (M)
Innovation (4 items)	Original or innovative product (D)
	Original or innovative product (M)
	Original or innovative product (G)
	Innovative fabrication/conservation process (G)
Local and traditional Production (12 items)	Local product (G)
	Proximity of production (G)
	Artisanal product (G)
	National production (G)
	Proximity of production (FV)
	Support for small-scale producers (G)
	Support for small-scale producers (M)
	Product with label (G)
	Food produced by cooperative (FV)
	Origin of production (M)
	Seasonal product (G)
	Traditional product (G)
Price (6 items)	Price of food (F)
	Price of food (D)
	Price of food (FV)
	Price of food (M)
	Price of food (G)
	Price-quality ratio (G)
Health (6 items)	Specific motivation for health issues (FV)
	Specific motivation for health issues (F)
	Nutritional composition (D)
	Health impact (G)
	Health impact (M)
	Nutritional composition (G)
Convenience (4 items)	Cooking convenience (M)
	Cooking convenience (FV)
	Cooking convenience (F)
	Cooking convenience (G)
*Second-order dimension*	
Healthy and environmentally friendly consumption	Ethics and Environment dimension
	Local and traditional production dimension
	Health dimension
	Absence of contaminants dimension

G: items constituting the part of the questionnaire regarding food choice motives in general; M: items constituting the part of the questionnaire regarding food choice motives for meat; FV: items constituting the part of the questionnaire regarding food choice motives for fruit and vegetables; D: items constituting the part of the questionnaire regarding food choice motives for dairy products; F: items constituting the part of the questionnaire regarding food choice motives for fish.

**Table 2:** Characteristics of the participants, NutriNet-Santé Study, *n* = 22,966 ^1^.

	Total Sample
*Sex (%)*	
Women	75.5
Men	24.5
*Age (year)*	53.7 ± 13.6
*Proportion of organic food in the diet* ^2^	0.28 ± 0.26
*Total energy intake (kcal/day)*	1989 ± 627
*Educational level (%)*	
<High school diploma	23.1
High school diploma	15.5
>High school diploma	61.4
*Monthly income per household unit (%)*	
Refuse to declare	12.5
900–1200 euros	10.6
1200–1800 euros	21.7
1800–2700 euros	25.7
>2700 euros	29.5
*Place of residence (%)*	
Rural community	22.0
Urban unit with a population smaller than 20,000 inhabitants	15.7
Urban unit with a population between 20,000 and 200,000 inhabitants	18.3
Urban unit with a population higher than 200,000 inhabitants	44.0

^1^ Values are means ± Standard Deviation (SD) or % as appropriate; ^2^ Ratios are computed by dividing the total organic food intake (g/day) out of the total intake excluding water (g/day).

**Table 3:** Mean values of the 32 variables (16 × 2 food group consumption) in grams per day included in the principal component analysis across clusters, NutriNet-Santé Study, *n* = 22,966 ^1^.

	Standard Conventional Food Small Eaters		Unhealthy Conventional Food Big Eaters		Standard Organic Food Small Eaters		Green Organic Food Eaters		Hedonist Moderate Organic Food Eaters	
*n* (%)	8819 (39%)		4405 (19%)		5983 (26%)		2640 (11%)		1119 (5%)	
Intake by food group (g/day)	Conventional food	Organic food	Conventional food	Organic food	Conventional food	Organic food	Conventional food	Organic food	Conventional food	Organic food
Fruits and vegetables (including juices and soups)	490.84 ± 293.19	96.67 ± 129.52	755.27 ± 424.55	89.64 ± 154.27	364.64 ± 265.78	385.69 ± 264.44	218.1 ± 235.03	762.53 ± 431.54	433.6 ± 275.69	205.24 ± 215.41
Seafood	32.91 ± 27.52	2.71 ± 6.76	57.09 ± 55.09	2.65 ± 8.35	35.02 ± 31.79	13.35 ± 18.64	34.1 ± 37.02	20.74 ± 29.5	46.07 ± 42.58	13.57 ± 29.01
Meat, poultry, processed meat	89.14 ± 52.75	9.43 ± 15.09	165.98 ± 100.03	10.84 ± 21.96	60.51 ± 47.67	36.75 ± 38.2	36.29 ± 42.6	47.11 ± 49.79	124.88 ± 84.79	31.97 ± 47.39
Eggs	5.01 ± 6.07	3.32 ± 5.32	10.67 ± 14.66	4.11 ± 7.56	2.18 ± 4.19	9.41 ± 9.78	1.04 ± 3.39	14.26 ± 14.96	5.91 ± 8.78	7.02 ± 9.79
Dairy products	216.34 ± 167.91	28.02 ± 58.83	310.69 ± 226.41	24.68 ± 62.93	124.99 ± 126.75	114.46 ± 128.07	50.52 ± 82.87	145.18 ± 176.81	142.17 ± 122.69	49.31 ± 75.59
Starchy foods	131.97 ± 77.91	16.43 ± 24.52	214.52 ± 118.96	15.94 ± 28.38	85.62 ± 70.08	62.82 ± 47.7	39.81 ± 54.62	135.82 ± 108.84	146.45 ± 90.82	38.03 ± 43.9
Whole grain products	30.67 ± 45.49	7.59 ± 19.44	39.91 ± 60.92	6.09 ± 19.07	26.78 ± 39.6	37.63 ± 47.62	13.48 ± 25.69	96.65 ± 94.02	34.71 ± 51.57	16.94 ± 36.99
Oil	11.46 ± 9.65	2.77 ± 5.12	21.29 ± 16.91	2.56 ± 6.01	7.28 ± 8.95	12.63 ± 11.35	3.04 ± 6.78	26.64 ± 19.09	12.91 ± 12.05	7.19 ± 9.73
Butter/Margarine	4.98 ± 4.9	0.56 ± 1.42	9.34 ± 8.79	0.58 ± 2.01	3.05 ± 4.19	2.83 ± 3.76	1.19 ± 2.98	4.94 ± 6.44	5.86 ± 6.12	1.76 ± 3.18
Sweetened foods	54.14 ± 39.72	5.93 ± 9.04	94.98 ± 74.83	6.17 ± 12.06	38.72 ± 32.7	23.24 ± 19.61	25.71 ± 28.46	45.64 ± 36.28	49.45 ± 37.69	13.85 ± 16.79
Alcoholic beverages	62.56 ± 79.29	4.24 ± 11.38	101.28 ± 121.14	3.72 ± 11.82	55.13 ± 65	17.63 ± 28.05	47.95 ± 74.88	46.85 ± 76.12	425.36 ± 337.49	92.46 ± 120.98
Non-alcoholic drinks	1543.08 ± 667.91	79.78 ± 149.54	1884.85 ± 856.68	62.11 ± 147.55	1409.18 ± 668.23	345.07 ± 322.37	1146.17 ± 628.26	682.93 ± 476.59	1446.15 ± 662.56	151.06 ± 225.59
Fast food	26.43 ± 21.02	1.38 ± 3.38	44.47 ± 49.86	1.47 ± 5.05	20.03 ± 20	8.15 ± 10.23	15.34 ± 40.91	19.03 ± 25.03	30.87 ± 26.53	5.28 ± 9.41
Extra food (including snacks, chips, salted biscuits, dressing and sauces)	10.61 ± 8.56	0.92 ± 2.5	22.75 ± 19.9	1.18 ± 4.1	8.98 ± 8.9	5.53 ± 7.51	6.39 ± 9.09	17.48 ± 18.48	15.36 ± 13.5	3.01 ± 5.07
Dairy and meat substitutes (including soy based products)	5.59 ± 29.72	6.57 ± 37.09	7.3 ± 38.43	5.05 ± 34.63	5.79 ± 23.27	23.16 ± 68.48	8.24 ± 49.72	95.81 ± 170.64	3.5 ± 18.21	6.18 ± 36.04
Other fats (including mayonnaise, fresh cream, vegetal fresh cream)	2.18 ± 2.32	0.22 ± 0.63	5.31 ± 7.11	0.32 ± 1.4	1.64 ± 2.29	1.26 ± 1.87	0.88 ± 2.28	3.58 ± 5.55	2.13 ± 2.6	0.57 ± 1.19

^1^ Values are means ± SD.

**Table 4:** Mean food choice motive dimension scores (scores ranging from 0 to 10), NutriNet-Santé Study, *n* = 22,966 ^1^.

Ranking	Dimension	Mean ± SD
1	Taste	8.90 ± 1.24
2	Health	7.68 ± 1.68
3	Absence of contaminants	7.66 ± 2.09
4	Local and traditional production	7.51 ± 1.73
5	Price	7.33 ± 1.91
6	Ethics and environment	5.85 ± 2.03
7	Convenience	5.48 ± 2.53
8	Innovation	3.53 ± 2.09
9	Avoidance for environmental reasons	2.79 ± 2.15
Second order dimension	Healthy and environmentally friendly consumption	7.17 ± 1.57

^1^ Values are means ± SD.

**Table 5:** Mean food choice dimension scores across clusters of consumers, NutriNet-Santé Study, *n* = 22,966 ^1,2^.

	Standard Conventional Food Small Eaters		Unhealthy Conventional Food Big Eaters		Standard Organic Food Small Eaters		Green Organic Food Eaters		Hedonist Moderate Organic Food Eaters	
	Mean	95% CI	Mean	95% CI	Mean	95% CI	Mean	95% CI	Mean	95% CI
Absence of contaminants	7.02 ^a^	(6.97–7.06)	6.84 ^b^	(6.79–6.90)	8.31 ^c^	(8.26–8.37)	8.94 ^d^	(8.87–9.02)	7.40 ^e^	(7.29–7.51)
Avoidance for environmental reasons	2.49 ^a^	(2.43–2.54)	2.24 ^b^	(2.17–2.3)	3.33 ^c^	(3.27–3.39)	4.15 ^d^	(4.07–4.24)	2.57 ^a^	(2.45–2.69)
Ethics and environment	5.25 ^a^	(5.2–5.29)	5.03 ^b^	(4.97–5.08)	6.52 ^c^	(6.47–6.57)	7.32 ^d^	(7.25–7.39)	5.67 ^e^	(5.57–5.78)
Taste	8.70 ^a^	(8.67–8.73)	8.77 ^b^	(8.73–8.80)	8.85 ^c^	(8.82–8.89)	8.89 ^c^	(8.84–8.94)	8.91 ^c^	(8.83–8.98)
Innovation	3.81 ^a^	(3.76–3.86)	3.83 ^a^	(3.77–3.90)	3.64 ^b^	(3.58–3.70)	3.32 ^c^	(3.24–3.41)	3.82 ^a,b^	(3.70–3.94)
Local and traditional production	6.96 ^a^	(6.93–7.00)	6.81 ^b^	(6.76–6.86)	7.98 ^c^	(7.94–8.03)	8.43 ^d^	(8.37–8.5)	7.40 ^e^	(7.31–7.50)
Price	7.67 ^a^	(7.63–7.72)	7.86 ^b^	(7.81–7.92)	7.09 ^c^	(7.04–7.14)	6.65 ^d^	(6.57–6.72)	7.27 ^e^	(7.16–7.38)
Health	7.33 ^a^	(7.29–7.37)	7.24 ^b^	(7.19–7.28)	7.88 ^c^	(7.83–7.92)	8.19 ^d^	(8.12–8.25)	7.12 ^b^	(7.03–7.22)
Convenience	5.65 ^a^	(5.58–5.71)	5.66 ^a^	(5.58–5.74)	5.08 ^b^	(5.00–5.15)	4.83 ^c^	(4.73–4.93)	5.15 ^b^	(5.00–5.31)
*Healthy and environmentally friendly consumption*	6.64 ^a^	(6.61–6.67)	6.48 ^b^	(6.44–6.52)	7.67 ^c^	(7.63–7.71)	8.22 ^d^	(8.17–8.28)	6.90 ^e^	(6.82–6.98)

^1^ Values are means (95% Confidence Intervals (CI)) adjusted for sex, age, educational level and household income; ^2^ Means annotated with the same letter are not different (*p* > 0.05), Tukey post-hoc test.

## Supplementary Materials

The following are available online at http://www.mdpi.com/2072-6643/9/2/88/s1, Figure S1: Food choice motives according to food categories across clusters.
