# Supplementary material for: Food Choice Motives When Purchasing in Organic and Conventional Consumer Clusters: Focus on Sustainable Concerns (The NutriNet-Santé Cohort Study)
Source: Nutrients. 2017 Jan 24;9(2):88. doi: 10.3390/nu9020088 (PMC5331519; doi:10.3390/nu9020088)
Supplement: Supplementary file 1 [file nutrients-09-00088-s001.docx]

Supplementary Materials: Food Choice Motives When Purchasing in Organic and Conventional Consumer Clusters: Focus on Sustainable Concerns (The NutriNet-Santé Cohort Study)

Julia Baudry, Sandrine Péneau, Benjamin Allès, Mathilde Touvier, Serge Hercberg, Pilar Galan, Marie-Josèphe Amiot, Denis Lairon, Caroline Méjean and Emmanuelle Kesse-Guyot

|  |  |
| --- | --- |
|  |  |
|  |  |

**Figure S1.** Food choice motives according to food categories across clusters.
